# A new *Maldane* species and a new Maldaninae genus and species (Maldanidae, Annelida) from coastal waters of China

**DOI:** 10.3897/zookeys.603.9125

**Published:** 2016-07-06

**Authors:** Yueyun Wang, Xinzheng Li

**Affiliations:** 1 Institute of Oceanology, Chinese Academy of Sciences, Qingdao 266071, China; 2 University of Chinese Academy of Sciences, Beijing 100049, China; 3 Laboratory for Marine Biology and Biotechnology, Qingdao National Laboratory for Marine Science and Technology, Qingdao, Shandong, 266070, China

**Keywords:** New species, new genus, Maldane, Paramaldane, Polychaeta, South China Sea, taxonomy

## Abstract

*Paramaldane*, new genus, with type species *Paramaldane glandicincta* **sp. n.**, and *Maldane adunca* **sp. n.** (Maldanidae, Polychaeta) are described based on material from the coast of south China. The new genus *Paramaldane* is similar to *Maldane* Grube, 1860 and *Sabaco* Kinberg, 1867, but it clearly differs from all genera within the subfamily Maldaninae by a unique combination of characters: the cephalic plate is almost circular with low, entire and smooth cephalic rim, nuchal grooves small and crescentic, lacking a collar on chaetiger 1, short companion notochaetae, a collar-like glandular band on the anterior part of the sixth chaetiger, and a well-developed anal valve. *Paramaldane glandicincta* **sp. n.** is characterised by having a glandular band on the anterior part of the sixth chaetiger, an almost circular cephalic plate, an entire and smooth cephalic rim, and small crescentic nuchal grooves. *Maldane adunca* **sp. n.** is characterised by a low cephalic rim, nuchal grooves with a strongly curved anterior part and isolated from the cephalic rim. Finally, a taxonomic key to genera of Maldaninae and a comparative table to species of *Maldane* are provided.

## Introduction

The Maldanidae, also known as bamboo worms, is a tubicolous and common family found in hard or soft substrates from the intertidal region to the deep sea (Paterson et al. 2009; De Assis and Christoffersen 2011). Maldanid species have a long, cylindrical body, generally with one or both truncate ends; elongated median segments with prominent tori on the end of each chaetiger; a keel-shaped prostomium fused to the peristomium; and a pair of nuchal grooves located on each side of the prostomium (Fauchald 1977; Fauchald and Rouse 1997; De Assis and Christoffersen 2011).

Arwidsson (1906) split Maldanidae into subfamilies after the major and complete revision of the family, leaving *Maldane* and *Asychis* in the nominotypical subfamily as the Maldaninae. The subfamily Maldaninae is recognised by the presence of cephalic and anal plates, and having the anus dorsal to the plate (Fauchald 1977). Light (1991) reviewed the Maldaninae and considered characters of cephalic and anal plates, the types of notochaetae, and the presence of a collar on chaetiger 1 as important generic characters. This author made a major revision of Maldaninae, and recognized six genera: *Asychis* Kinberg, *Maldane* Grube, *Sabaco* Kinberg, *Bathyasychis* Detinova, *Chirimia* Light and *Metasychis* Light. Posteriorly, De Assis and Christoffersen (2011) analyzed the phylogenetic relationships within Maldanidae based on morphological characters.

During a sorting of the Maldanine specimens deposited in the Marine Biological Museum, Chinese Academy of Sciences
(MBM) in the Institute of Oceanology, Chinese Academy of Sciences, Qingdao (IOCAS), some specimens of *Asychis*-like species were identified, which belonged to an unknown species. Based on these specimens, two new species are fully described and illustrated and a new genus of Maldaninae is proposed. A taxonomic key to genera of Maldaninae and a table comparing the morphology of all species of *Maldane* are provided.

## Material and methods

The specimens were collected from the South China Sea from 1959 to 1962. They have been stored in 70% ethanol. Specimens were examined under Zeiss Stemi 2000-C stereomicroscopes, and compound microscopes. Drawings were prepared with the aid of ‘AxioCam MRc 5’digital camera fitting on the stereomicroscopes. Line drawings are completed in the Adobe Photoshop CS6 using a graphics tablet. Notochaetae and neurochaetae were extracted carefully and observed under optical and scanning electron microscopes (SEM). All specimens are deposited in the Marine Biological Museum, Chinese Academy of Sciences
(MBM). An identification key to the genera of Maldaninae modified from Light (1991) is provided below. Table 1 compares morphological characters for all known species of genus *Maldane*.

**Table 1.** Morphological comparison of species of *Maldane*. Unless otherwise indicated, character information is from Light (1991) and the original descriptions and illustrations. Unknown information marked with ‘?’.

Characters	*Maldane adunca* sp. n.	*Maldane arctica* Detinova, 1985	*Maldane californiensis* Green, 1991	*Maldane capensis* (Day, 1961)	*Maldane cristata* Treadwell, 1923	*Maldane cuculligera* Ehlers, 1887
Type locality	Southwest of Macao	Arctic	Southern California	South Africa	California	Gulf of Mexico
Collar on chaetiger 1	No	No	Yes, limited ventral side	No	No	Yes, limited ventral side
Pigmentation	absent	?	?	Head of living worm flecked with brown	Anterior segments with dark brown pigment	Nuchal groove with brown pigment spot?
Shape of nuchal grooves	Strongly curved anteriorly, J-shaped	Short, slightly curved	Slightly curved	J-shaped	Short and divergent anteriorly	slightly curved
Posterior cephalic rim	Low	Pocket-like	Pocket-like	Pocket-like	Pocket-like	Pocket-like
Dorsal glandular band on chaetiger 5	Absent	?	Absent	Absent	Sixth chaetiger with an anterior dorsal flange*	Dorsal glandular band
Prostomial palpode	Bluntly rounded	Spade-like	Rounded to semi-triangular	Broadly spatulate	Hemispherical	Bluntly rounded
Border of anal plate	Laterally notched	Laterally notched	Laterally notched	Laterally notched	Laterally notched	Laterally notched
Ventral part of anal rim	Smooth to slightly crenulate	Smooth	Slightly crenulate	Crenulated	Slightly crenate	Smooth to slightly crenulate
Characters	*Maldane decorata* Grube, 1877**	*Maldane glabra* Knox & Cameron, 1971	*Maldane glebifex* Grube, 1860	*Maldane gorgonensis* Monro, 1933	* Maldane malmgreni* McIntosh, 1885***	* Maldane marsupialis* Grube, 1878
Type locality	Congo	Port Phillip Bay, Australia	French	Gorgona Island, Colombia	Strait of Gibraltar	Philippines
Collar on chaetiger 1	No	Ventrally; inconspicuous	No	No	No	No
Pigmentation	?	?	No pigmentation	?	?	2 eye spots on peristomium
Shape of nuchal grooves	?	Faintly J-shaped	Short and arched	Boldly curved	?	Slightly curved
Posterior cephalic rim	?	Pocket-like	Pocket-like	Pocket-like	Low	Pocket-like
Dorsal glandular band on chaetiger 5	?	Absent	Absent		?	Absent
Prostomial palpode	?	Prow-like	Spade-like	Bluntly rounded	?	Spade-like
Border of anal plate	?	Laterally notched	Laterally notched	Complete, no notches	?	Complete, no notches
Ventral part of anal rim	?	Smooth	Crenulated	Smooth	?	Smooth
Characters	* Maldane meridionalis* (Chamberlin, 1919)	* Maldane monilata* Fauchald, 1972	* Maldane philippinensis* Treadwell, 1931	* Maldane pigmentata* (Imajima & Shiraki, 1982)	* Maldane sarsi* Malmgren, 1865	* Maldane theodori* Augener, 1926
Type locality	Between Galapagos Islands and Peru	Middle America Trench	Darvel Bay	Kashima Sea	Sweden	Queen Charlotte Sound, New Zealand
Collar on chaetiger 1	No	Yes	No	No	No	Yes
Pigmentation	Living worm with dark pigment areas on anterior body	Without distinct color patterns	?	Anterior body with brown spots	Anterior end with black-brown pigmentation, but smaller individuals may be missing	?
Shape of nuchal grooves	Short, curved	Short, curved	Short, curved	Short, curved in a semicircle	Short, slightly curved	J-shaped
Posterior cephalic rim	?	Pocket-like	Pocket-like	Low	Pocket-like	Pocket-like
Dorsal glandular band on chaetiger 5	Absent	Absent	?	Absent	Crescentic glandular band, but not always present	?
Prostomial palpode	Narrow and pointed	Broudly rounded	Spade-like	Broudly rounded	Spade-like	Broadly spatulate
Border of anal plate	?	Laterally notched	Laterally notched	Laterally notched	Laterally notched	Laterally notched
Ventral part of anal rim	?	Crenulated	Smooth	Smooth	Smooth to slightly crenulated	Crenulated

* from Imajima and Shiraki 1982.

** *Maldane decorata* Grube, 1877 inadequate descriptions and no illustrations in original paper.

*** *Maldane malmgreni* McIntosh, 1885, inadequate descriptions in original paper.

## Systematics

### Family Maldanidae Malmgren, 1867 Subfamily Maldaninae Malmgren, 1867

#### Genus Paramaldane gen. n.

Taxon classificationAnimaliaCapitellidaMaldanidae

http://zoobank.org/DE537EA3-4C8C-485F-9684-23D70FF5229E

##### Type species.

*Paramaldane glandicincta* sp. n.

##### Diagnosis.

Body with19 chaetigers. Cephalic plate circular. Prostomial palpode bluntly rounded, and confluent with cephalic rim. Cephalic rim low and entire with slight incisions. Cephalic keel short. First chaetiger without collar. Chaetiger 6 with a collar-like glandular band. Neurochaetae beginning to present on the second chaetiger. Notochaetae spirally fringed with short companion chaetae. Two preanal achaetigerous segment. Anus dorsal, with anal valve. Anal plate well-developed, but no anal cirri; with two lateral deep incisions on anal rim.

##### Etymology.

The generic name is a combination of the prefix para- (meaning resembing) and the generic name *Maldane*. The new genus is related to *Maldane* in morphology. Gender: feminine.

##### Remarks.

The new genus *Paramaldane* is superficially similar to *Maldane* Grube, 1860 and *Sabaco* Kinberg, 1867. The anal plate and notochaetae type of *Paramaldane* are closer to *Maldane*. The shape of prostomial palpode and nuchal grooves are closer to *Sabaco*. However, the new genus can be easily distinguished by the characters of the cephalic plate, which are considered to be of generic importance (Light 1991; Green 1994). The cephalic rim of *Maldane* and *Sabaco* is divided into two lateral lobes and a posterior lobe by deep lateral notches, but that of the *Paramaldane* is almost smooth. The prostomial palpode of *Maldane* is spade-like, but that of *Paramaldane* is bluntly rounded and confluent with cephalic rim. Both *Sabaco* and *Paramaldane* have small crescentic nuchal grooves that are isolated from cephalic rim, but *Sabaco* has a complete collar on the first chaetiger that is lacking in *Paramaldane*. Notochaetae of *Sabaco* have long companion chaetae, but companion notochaetae of *Paramaldane* are short. An identification key to the genera of Maldaninae modified from Light (1991) is provided below.

#### Paramaldane glandicincta sp. n.

Taxon classificationAnimaliaCapitellidaMaldanidae

http://zoobank.org/B4BB3FC1-50B5-4A56-B9D5-83B84F894F63

Figs 1 , 2

##### Type material examined.

Holotype: MBM 008120, complete. Original label: South China Sea, Station 6175, mud sediment, 141 m, 28 January 1959. Paratypes: MBM 008130, 1 complete specimen, Southeast of Hainan Island, 18°30'N, 110°45'E. Original label: South China Sea, Station 6156, mud sediment, 100 m, 8 March 1960; MBM 008214, two incomplete specimens, posterior part lost, Southeast of Hainan Island, 18°30'N, 110°30'E. Original label: South China Sea, Station 6143, mud sediment, 122.5 m, 22 April 1959.

##### Type locality.

China, south of Hainan Island, 17°30'N, 110°00'E, 28 January 1959.

##### Diagnosis.

Complete specimen with 19 chaetigers and two preanal achaetigerous segments. Cephalic plate rounded. Cephalic rim with two lateral creases, margin of the rim almost smooth. Anterior chaetigers biannulate. Sixth chaetiger with thick, collar-like glandular band. Rim of anal plate with deep lateral notches,ventral margin of anal rim crenulate, dorsal margin smooth.

##### Description.

**Figure 1.**
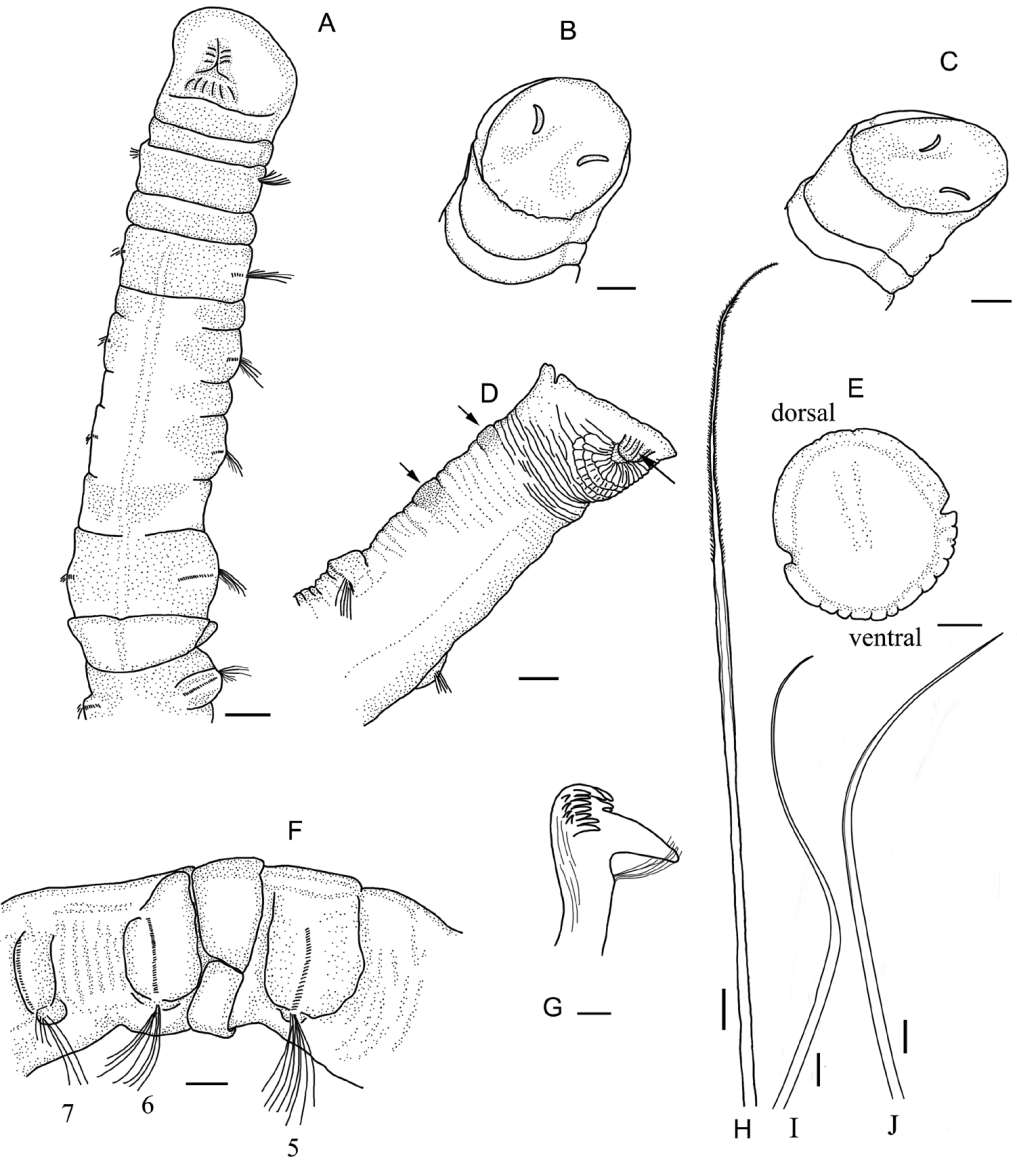
*Paramaldane glandicincta* sp. n. **A** ventral side of anterior body **B** dorsal view of cephalic plate **C** lateral view of cephalic plate **D** ventral view of pygidium, arrows show preanal achaetigerous segments and anal valve **E** frontal view of anal plate **F** lateral view of glandular band on sixth chaetiger, showing collar-like glandular band **G** lateral view of neurochaeta from chaetiger 5 **H** spirally-fringed notochaeta from chaetiger 10 **I** geniculate companion chaeta from chaetiger 10 **J** capillary companion chaeta from chaetiger 10. Scale bars: **A–F** = 0.5 mm, **G** = 10 μm, **H** = 50 μm, **I–J** = 200 μm.

**Figure 2.**
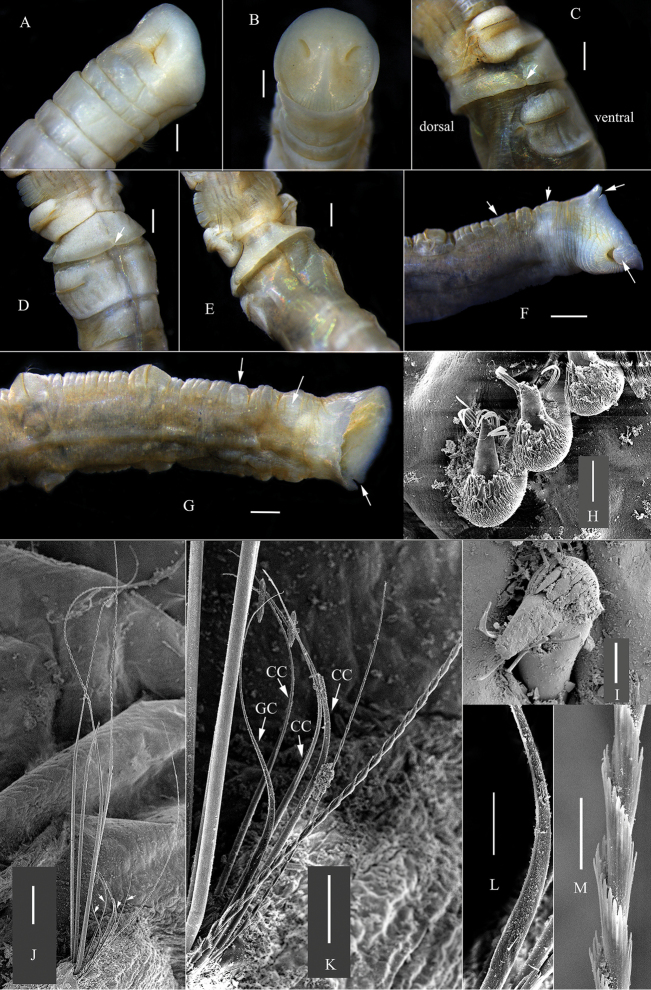
*Paramaldane glandicincta* sp. n. **A–G** holotype of MBM 008120 **A** ventral side of anterior end **B** cephalic plate **C** lateral view of glandular band, arrow shows lateral slit **D** ventral side of glandular band, arrow shows the midventral notch **E** dorsal side of glandular band **F–G** dorsal and ventral side of pygidium, arrows show preanal achaetigerous segments, anal valve and lateral notch on anal plate **H–M** chaetae from MBM 008214 **H–I** neurochaetae from 8^th^ and 4^th^ chaetigers respectively; **J** notochaetae from 8^th^ chaetiger, arrows show companion chaetae **K** companion chaetae from 8^th^ chaetiger **L** transitional part of geniculate capillary **M** spinose part of notochatae. GC, geniculate companion chaeta. CC, capillary companion chaeta. Scale bars: **A–G** = 0.5 mm, **H–I** = 100 μm, **J** = 1.0 mm, **K** = 500 μm, **L** = 200 μm, **M**=100 μm.

Holotype complete, 43 mm long, and 2.0 mm wide at the third chaetiger. Paratype of MBM 008130 complete, 74 mm long and 2.5 mm wide. Body cylindrical with 19 chaetigers, two preanal achaetigerous segments, and pygidium. First chaetiger without neurochaetae. Anterior part of the sixth chaetiger with thick glandular band forming a low collar and overlapping posterior part of the fifth chaetiger (Figs 1A, F; 2C–E).

Cephalic plate obliquely truncated, edge almost circular (Fig. 1B, C). Cephalic rim smooth (Fig. 1C), with a pair of shallow lateral creases (Fig. 1B). Deep furrow from lateral crease runs backward on peristomium to front edge of first chaetiger. Margin of posterior part of cephalic rim very weakly undulating (Fig. 1B, C). Anterior parts of rim completely smooth, and fused with prostomial palpode. Prostomial palpode indistinct, smoothly circular. Cephalic keel short and slightly arched. Nuchal grooves short, slightly curved, isolated from cephalic rim (Fig. 1B).

First four chaetigers completely biannulate, each comprising an achaetigerous and chaetigerous annulus. First six chaetigers short, following chaetigers elongated. Epidermal glands developed well on chaetigers 1–6. Glands only present on parapodial tori of following segments.Thick glandular band resembling a collar located on anterior part of sixth chaetiger, covering rear of fifth chaetiger, divided into dorsal and ventral parts by two lateral slits (Figs 1F, 2C). Dorsal margin of glandular band smooth (Fig. 2E). A small notch on ventral median line of ventral glandular band (Figs 1A, 2D).

Neurochaetae beginning to present on second chaetiger, with many small teeth on main fang (Figs 1G, 2H, I). Anterior chaetigers with simple capillary notochaetae . Middle and posterior chaetigers with long spirally-fringed notochaetae and short companion notochaetae (Fig. 2J). Long notochaetae with spinose spiral bands imbricated over main shaft (Figs 1H, 2M). Short companion chaetae two kinds: geniculate and capillary chaetae (Figs 1I, J, 2K). Geniculate companion chaetae with a long whip-like tip (Fig. 2K); transitional part smooth and thicker than shaft (Fig. 2L).

Two preanal achaetigerous segments marked by parapodial rudiments (Figs 1D, 2F, G). First achaetigerous segment longer than last one. Anus on dorsal side with a flaplike anal valve (Figs 1D, 2F). Pygidium forming a flat anal plate with a pair of deep lateral notches (Fig. 2F). Ventral part of the anal rim with 7–8 conspicuous crenulations (Fig. 1E). Dorsal rim smooth to slightly crenulated.

##### Etymology.

The specific name *glandicincta* is a combination of *glans* and *cinctus* (meaning "belt", feminine form *cincta*), refering to the characteristic glandular belt on the six chaetiger.

##### Remarks.

*Paramaldane glandicincta* sp. n. is characterised by a collar-like glandular band on the anterior margin of the sixth chaetiger.

#### Genus *Maldane* Grube, 1860

##### Maldane adunca sp. n.

Taxon classificationAnimaliaCapitellidaMaldanidae

http://zoobank.org/B3061C48-1D4E-4140-808D-771F70BADAB6

Figs 3 , 4 , 5

###### Type material examined.

Holotype: MBM 008111, complete. Original label: South China Sea, Station 6076, mud sediment, 39 m, 21 April 1959. Paratypes, same collecting data as holotype, MBM 240860–240861, nine specimens.

Other material examined: MBM 008125, 1 complete specimen, south of Macao, 21°30'N, 113°30'E, Station 6062, silt sediment, 35 m, 24 April 1959; MBM 006330, 10 complete specimens, northeast of Hainan Island, 20°00'N, 111°30'E, Station 6119, mud sediment, 70 m, 12 April 1959; MBM 201498, 1 anterior part, Beibu Gulf, Station 6209, mud sediment, 56.8 m, 6 July 1960; MBM 201496, 1 complete worm, Beibu Gulf, Station 7905, silt sediment, 29 m, 1 January 1962; MBM 201494,1 complete worm, Beibu Gulf, Station 6200, mud sediment, 32.5 m, 13 July 1960.

###### Type locolity.

China, southwest of Macao, 21°00'N, 113°00'E, 21 April 1959 .

###### Comparative material examined.

***Maldane sarsi*.** MBM 241068, 2 complete specimen, west of Point Barrow, 71°29.170'N, 161°58.899'W, Station C17, mud sediment, 45 m, 8 August 2008; MBM 008150, 2 complete specimen, north of Yantai, Shandong Province, 38°06'N, 121°31.98'E, Station 2009, mud sediment, 57.5 m, 18 October 1958; MBM 008062, 1 complete specimen, the Yellow Sea, 36°30'N, 124°00'E, Station 3022, mud sediment, 70 m, 21 January 1959; MBM 008228, 1 complete specimen, east of Zhoushan Islands, 29°45'N, 122°30'E, Station 4128, mud sediment, 54 m, 12 July 1959; MBM 008009, 1 complete specimen, the East China Sea, 28°30'N, 123°30'E, Station 4074, mud sediment, 77 m, 9 December 1959; MBM 201497, 1 complete specimen, west of Hainan Island, 18°35.36'N, 106°50.58'E, Station 7702, mud sediment, 55 m, 20 January 1962;

###### Diagnosis.

Cephalic plate obliquely truncated, elliptical. Cephalic rim low and divided into lateral and dorsal lobes by lateral incisions. Lateral cephalic rim confluent with prostomial palpode. Prostomial palpode bluntly rounded. Nuchal grooves deep and strongly curved outward anteriorly, J-shaped. Anal plate almost truncate and rounded. Rim of anal plate low, with deep lateral incisions.

###### Description.

**Figure 3.**
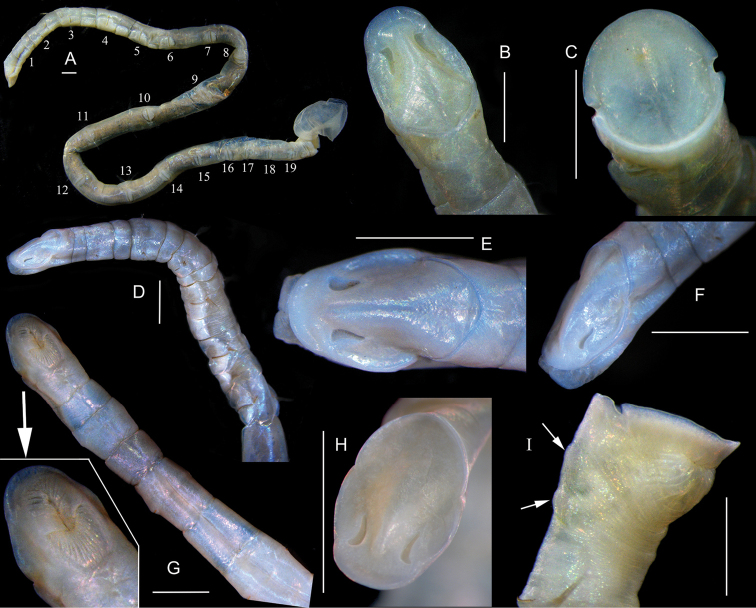
*Maldane adunca* sp. n. **A–C** paratype of MBM 240860 **A** complete body of MBM 240860 **B** dorsal view of cephalic plate **C** end view of anal plate D–F, paratype of MBM 240861 **D** anterior body showing glandular pads **E** dorsal view of cephalic plate **F** lateral view of cephalic plate **G–I** MBM 006330 **G** ventral view of anterior body **H** dorsal view of cephalic plate **I** pygidium, arrows shows pre-anal achaetigerous segments. Scale bars: 0.5 mm.

**Figure 4.**
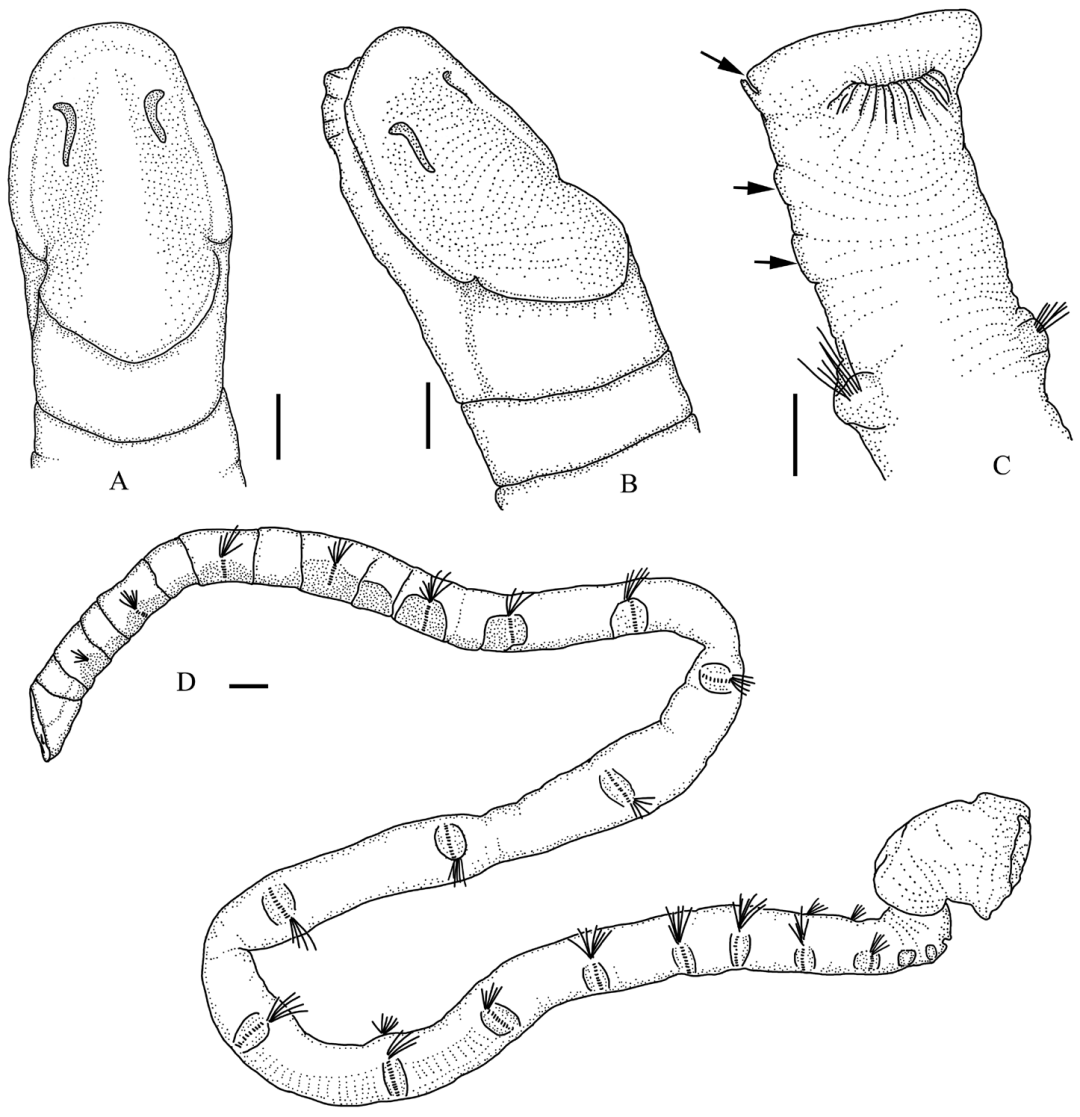
*Maldane adunca* sp. n. **A** dorsal view of cephalic plate **B** lateral view of cephalic plate **C** dorsal view of pygidium, arrows show pre-anal achaetigerous segments and lateral notch of rim of anal plate **D** complete body. Scale bars = 0.5mm.

Holotype about 65 mm in length, 1.5 mm in width. Largest specimen more than 70 mm in length, and 3.0 mm in width. Segments short on anterior and posterior body, longer on middle body (Figs 3A, 4D).

Body with 19 chaetigers, two preanal achaetigerous segments followed by a pygidium. Cephalic plate obliquely truncated, elliptical (Figs 3B, E, H, 4A). Prostomial palpode bluntly rounded, perfectly fused with cephalic rim. Cephalic rim lower and smooth, with two lateral notches. Cephalic keel remarkable, high and long, with posterior part widens (Figs 3B, E, 4A, B). Nuchal grooves short, anteriorly strongly curved outward, J-shaped (Figs 3B, E, H, 4A). Nuchal grooves isolated from cephalic rim. Mouth trilobed, and divided into upper and lower lips by a transverse fissure. Upper lip incised medially (Fig. 3G).

**Figure 5.**
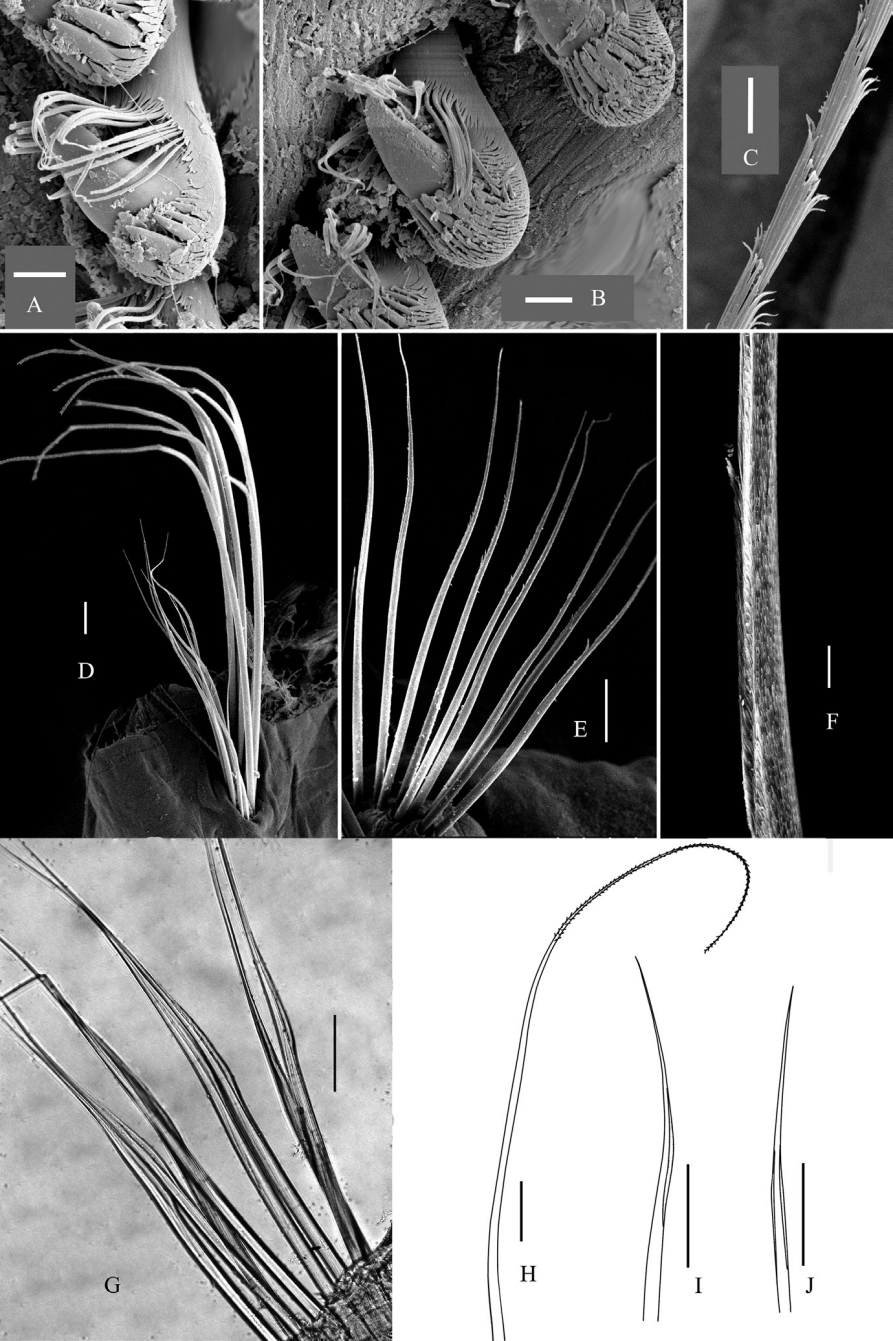
Chaetae of *Maldane adunca* sp. n. **A–B** neurochaetae from the 2^nd^ and 17^th^ chaetigers **C** spinose part of notochaetae **D** notochaetae from 16^th^ chaetiger **E** short limbate companion chaetae from 18^th^ chaetiger **F** transitional part of geniculate companion chaetae **G** companion chaetae from 14^th^ chaetiger. H–J, notochaetae drawn from optical microscope **H** spirally-fringed notochaetae **I** geniculate companion chaetae **J** bilimbate companion chaetae. BC, bilimbate companion chaeta. GC, geniculate companion chaeta. Scal bars: **A–C** = 60 μm, **D–E** = 0.5 mm, **F–G** = 50 μm, **H–J** = 0.5 mm.

First five chaetigers biannulate (Figs 3A, D, 4D). First chaetiger without neurochaetae. Neurochaetae typical rostrate uncini similar on all chaetigers (Fig. 5A, B). Neurochaeta with several transversal rows of small teeth on main fang. Anterior chaetigers with capillary notochaetae. Middle and posterior chaetigers with spirally fringed notochaetae (Fig. 5D, H); spinose spiral bands closely imbricated over main shaft (Fig. 5C). Short companion chaetae geniculate (Fig. 5F, J, I), narrowly limbate (Fig. 5E) and bilimbate (Fig. 5G, J).

Two short and rudimentary preanal achaetigerous segments (Figs 3I, 4C), which deeply stained with methyl green. Anal pore with a less-developed anal valve (Fig. 3I). Anal mound well developed. Anal plate truncated, nearly rounded; median part of plate with a shallow furrow dorso-ventrally extended (Fig. 3C). Rim of anal plate low and incised laterally (Fig. 3C, I). Dorsal part of rim smooth. Ventral part of the rim smooth to weakly serrated.

###### Variation.

Body wall of small individuals thin but thick in large ones. Body of small individuals smooth, semitransparent and lacking epidermal glands. Large individuals with glandular pads on parapodial tori and ventral side of chaetigers 3–5.

###### Etymology.

The specific epithet is the Latin adjective *adunca* (feminine, meaning hooked) and refers to the strongly curved nuchal grooves.

###### Remarks.

*Maldane adunca* sp. n. is distinctive in the genus *Maldane* with its low cephalic rim and hook-like nuchal grooves. *Maldane adunca* sp. n. is close to *Maldane sarsi* Malmgren, 1865, a potential species-complex, which is thought to be a cosmopolitan species (Day 1967, Hartman 1961). However, the new species differs from the latter by possessing a low cephalic rim, strongly curved nuchal grooves which are isolated from the cephalic rim, and lacking crescentic glandular bands on the dorsal surface of the fifth chaetiger. In *Maldane sarsi*, the cephalic rim is well developed, its posterior part forms a deep pocket-like structure (Arwidsson 1906) and overlaps the posterior part of cephalic keel, cephalic keel is strongly arched, the nuchal grooves are narrow and slightly curved and connected with margin of cephalic rim, and the dorsal surface of the fifth chaetiger sometimes bears a crescentic glandular band (Green 1991, Fauvel 1953). *Maldane adunca* sp. n. is also closely related to *Maldane glebifex* Grube, 1860. The new species differs from the latter in the form of the anal rim and nuchal grooves. *Maldane glebifex* has a crenulated border to the anal plate while *Maldane adunca* sp. n. has a smooth to slightly crenulated anal rim. The nuchal grooves of *Maldane adunca* sp. n. are much more curved than that of *Maldane glebifex*. In terms of geographical distribution, *Maldane glebifex* is a Mediterranean/North Atlantic species (Fauvel 1927), and it is unlikely to occur in the South China Sea.

Light (1991) revised the subfamily Maldaninae and recognized 16 species of *Maldane*, of which *Maldane pellucida* Sars, 1869 was recognized later as *nomina nuda* (Oug et al. 2014). At present, *Maldane* includes 18 species: *Maldane adunca* sp. n., *Maldane arctica*, *Maldane californiensis*, *Maldane capensis*, *Maldane cristata*, *Maldane cuculligera*, *Maldane decorate*, *Maldane glabra*, *Maldane glebifex*, *Maldane gorgonensis*, *Maldane malmgreni*, *Maldane marsupialis*, *Maldane meridionalis*, *Maldane monilata*, *Maldane philippinensis*, *Maldane pigmentata*, *Maldane sarsi*, *Maldane theodori*. *Maldane sarsi* includes two subspecies: *Maldane sarsi antarctica* Arwidsson, 1911 and *Maldane sarsi borealis* Imajima, 1963 but their validity is doubtful. *Maldane sarsi antarctica* resembles the stem species. Color and gland pattern is main difference between the subspecies and its stem species according to Arwidsson (1911), but they are not robust taxonomic characters. Imajima (1963) collected only one specimen to erect *Maldane sarsi borealis*. This subspecies has 18 chaetigers, and anal plate of it incised ventrally. The chaetiger number is unusual in *Maldane* (usually, 19 chaetigers in *Maldane* species). Table 1 compares morphological characters for all known species of genus *Maldane*.

#### Key to the genera of Maldaninae

**Table:** 

1	First two chaetigers without neurochaetae	***Bathyasychis* Detinova, 1982**
–	Only first chaetiger without neurochaetae	**2**
2	Chaetiger 6 without collar-like glandular band	3
–	Chaetiger 6 with collae-like glandular band	***Paramaldane* gen. n.**
3	Pygidium with anal valve	**4**
–	Pygidium without anal valve	**5**
4	Nuchal grooves U-shaped; prostomial palpode mushroom-shaped	***Chirimia* Light, 1991**
–	Nuchal grooves slightly curved to J-shaped; prostomial palpode spade-like	***Maldane* Grube, 1860**
5	First chaetiger without a collar	***Asychis* Kinberg, 1867**
–	First chaetiger with a collar complete or limited to the ventral side	**6**
6	Nuchal grooves J- or U-shaped; prostomial palpode mushroom-shaped; cephalic rim with crenulations or digitiform cirri; first chaetiger with a collar usually ventrally limited, sometimes complete	***Metasychis* Light, 1991**
–	Nuchal grooves small, crescentic; prostomial palpode spadelike or indistinct; cephalic rim smooth without crenulations or digitiform cirri; the first chaetiger with a complete collar	***Sabaco* Kinberg, 1867**

## Supplementary Material

XML Treatment for Paramaldane

XML Treatment for Paramaldane glandicincta

XML Treatment for Maldane adunca

## References

[B1] Arwidsson I (1907) Studien über die Skandinavischen und Arktischen Maldaniden nebst zusammenstellung der übrigen bisher bekannten Arten dieser Familie. Zoologische Jahrbcher 9(Suppl.): 1–308.

[B2] Arwidsson I (1911) Die Maldaniden. Wissenschaftliche Ergebnisse der Schwedischen Südpolar Expedition 1901-1903 Stockholm Zoologie II, Band 6, Lieferung 6: 1–44.

[B3] Augener H (1926) Papers from Dr. Th. Mortensen’s Pacific Expedition 1914–16 – XXXIV Polychaeta III – Polychaeten von Neuseeland – II Sedentaria. Videnskabelige Meddelelser fra Dansk naturhistorisk Forening i Köbenhavn 81: 157–294.

[B4] Chamberlin RV (1919) The Annelida Polychaeta. Museum of Comparative Zoölogy at Harvard College 48: 1–514.

[B5] Day JH (1961) The polychaeta fauna of South Africa. Pt. 6. Sedentary species dredged off Cape coasts with a few new records from the shore. Journal of the Linnean Society London 44: 463–560. doi: 10.1111/j.1096-3642.1961.tb01623.x

[B6] Day JH (1967) A monograph on the Polychaeta of southern Africa. Part 2. Sedentaria. British Museum of Natural History Publications 656: 459–878.

[B7] De Assis JE Christoffersen ML (2011) Phylogenetic relationships within Maldanidae (Capitellida: Annelida), based on morphological characters. Systematics and Biodiversity 9: 41–55. doi: 10.1080/14772000.2011.604358

[B8] Detinova NN (1985) O taksonomicheskom znachennii stroenyia parapodii u nekotorykh Maldanidae (Polychaeta). [On the taxonomic significance of the structure of parapodia in some Maldanidae (Polychaeta)]. Zoologichesky Zhurnal 64: 1487–1492. [In Russian with English summary]

[B9] Ehlers E (1887) Report on the annelids of the dredging expedition of the US coast survey steamer ‘Blake”. Memoirs of the Museum of Comparative Zoology, Harvard 15, 335 pp.

[B10] Fauchald K (1972) Benthic polychaetous annelids from deep water off western Mexico and adjacent areas in the Eastern Pacific Ocean. Allan Hancock Monographs in Marine Biology 7: 1–575.

[B11] Fauchald K (1977) The polychaete worms, definitions and keys to the orders, families and genera. Natural History Museum of Los Angeles County, Science Series 28: 1–188.

[B12] Fauchald K Rouse G (1997) Polychaete systematics: past and present. Zoologica Scripta 26(2): 71–138. doi: 10.1111/j.1463-6409.1997.tb00411.x

[B13] Fauvel P (1927) Polychètes sédentaires. Addenda aux errantes, Archiannélides, Myzostomaires. Faune de France, Paris, 494 pp.

[B14] Fauvel P (1932) Annelida Polychaeta of the Indian Museum, Calcutta. Memoirs of the Indian Museum 12: 1–262.

[B15] Fauvel P (1953) The Fauna of India including Pakistan, Ceylon, Burma and Malaya. AnnelidaPolychaeta. Indian Press, Allahabad, 507 pp.

[B16] Green KD (1991) *Maldane californiensis*, a new species (Polychaeta: Maldanidae) and a review of its relations. Bulletin of Marine Science 48(2): 214–226.

[B17] Green KD (1994) The head of Maldanidae polychaetes of the subfamily Maldaninae. Memoires du Museum National d’Histoire Naturelle 162: 101–109.

[B18] Grube AE (1860) Beschreibung neuer oder wenig bekannter Anneliden. Archiv für naturgeschichte 26: 71–118.

[B19] Grube AE (1877) Anneliden - Ausbeute S.M.S. Gazelle. Monatsbericht der Koniglich Preussischer Akademie der Wissenschaften zu Berlin, 509–554.

[B20] Grube AE (1878) Annulata Semperiana. Beitrãge zur Kenntnis der Annelidenfauna der Philippinen nach den von Herrn Prof. Semper mitgebrachten Sammlungen. Mem. Acad. Sci. St. Petersburg 25: 1–300

[B21] Hartman O (1961) Polychaetous annelids from California. Allan Hancock Pacific Expeditions 25: 1–226.

[B22] Imajima M (1963) Polychaetous annelids collected off the west coast of Kamchatka II. Notes on species found in the collection of 1959. Publications of the Seto Marine Biological Laboratory 11(2): 345–372.

[B23] Imajima M Shiraki Y (1982) Maldanidae (Annelida: Polychaeta) from Japan. (Part 2). Bulletin of the National Science Museum, series A (Zoology) 8(2): 47–88

[B24] Kinberg JH (1867) Annulata nova. Öfversigt af Kongliga Vetenskaps-Akademiens förhandlingar, Stockholm 23: 337–357.

[B25] Knox GA Cameron DB (1971) Port Phillip Bay Survey Part 2. Polychaeta. Memoirs of the National Museum of Victoria 32: 21–41.

[B26] Light WHJ (1991) Systematic revision of the genera of the Polychaeta subfamily Maldaninae Arwidsson. Ophelia Supplement 5: 133–146.

[B27] Malmgren AJ (1865) Nordiska Hafs-Annulater. Öfversigt af Königlich Vetenskaps-A kademiens Förhandlingar 22: 181–192.

[B28] Malmgren AJ (1867) Annulata Polychaeta Spetsbergiae, Gröenlandiae, Islandiae et Scandinaviae hactenus cognita. Ex Officina Frenckelliana, Helsingforslae, 127 pp. doi: 10.5962/bhl.title.13358

[B29] McIntosh WC (1885) Report on the Annelida Polychaeta collected by H.M.S. Challenger during the years 1873–76. Challenger Report 12: 1–554.

[B30] Monro CCA (1933) The Polychaeta Sedentaria collected by Dr. C. Crossland at Colón, in the Panama Region, and the Galapagos Islands during the Expedition of the S.Y. ‘St. George’. Proceedings of the Zoological Society of London 103(4): 1039–1092. doi: 10.1111/j.1096-3642.1933.tb01640.x

[B31] Oug E Bakken T Kongsrud JA (2014) Original specimens and type localities of early described polychaete species (Annelida) from Norway, with particular attention to species described by OF Muller and M. Sars. Memoirs of Museum Victoria 71: 217–236.

[B32] Paterson GL Glover AG Froján CB Whitaker A Budaeva N Chimonides J Doner S (2009) A census of abyssal polychaetes. Deep Sea Research Part II: Topical Studies in Oceanography 56(19): 1739–1746. doi: 10.1016/j.dsr2.2009.05.018

[B33] Treadwell AL (1923) Polychaetous Annelids from Lower California with descriptions of new species. American Museum Novitates 74: 1–11.

[B34] Treadwell AL (1931) Contributions to the biology of the Philippine Archipelago and adjacent regions. Four new species of polychaetous annelids collected by the United States fisheries steamer ‘Albalross’ during the Philippine Expedition of 1907-1910. Bulletin of the United States National Museum 100: 313–321.

